# Rethinking drug safety signal detection and causality assessment in the age of AI: the risks of incomplete data and biased insights 

**DOI:** 10.3389/fdsfr.2025.1678074

**Published:** 2025-09-16

**Authors:** Priyanka Chhikara, Tarek A. Hammad

**Affiliations:** ^1^ Global Safety Science, Global Safety and Pharmacovigilance, CSL Behring, King of Prussia, PA, United States; ^2^ Medical Safety of Marketed Products Development and Plasma-Derived Therapies, Patient Safety and Pharmacovigilance, Takeda Development Center Americas, Inc., Cambridge, MA, United States

**Keywords:** pharmacovigilance, artificial intelligence, missing data, signal detection, causality assessment

## Introduction

Artificial Intelligence (AI) is a term used for systems that can perform humanlike cognitive functions like learning, perception, interpretation and problem solving. AI systems learn and improve by analyzing large datasets, and they are powered by algorithms, computing power, and specialized hardware. However, introduction of AI in healthcare comes with its own biases and disparities. There is emerging evidence that the Artificial Intelligence (AI) models do not transcend bias–in fact they learn to inherit it. The models seem to accept missing data and its structural inequities to shape what they see–rather what they do not see. In the world of signal detection and assessment, such data inadequacies can pose much more than a simple technical flaw. This paper investigates the challenges and implications of adopting AI when its promise is matched by its shortcomings.

There is emerging evidence that these systems may not only reflect clinical knowledge but also reproduce or even amplify societal biases when generating medical recommendations. The recent study by Omar et al. (2025) provides an evaluation of sociodemographic biases in clinical recommendations generated Artificial Intelligence (AI) tools. By analyzing over 1.7 million outputs across nine models using standardized emergency department cases, the authors identified consistent and clinically unjustified differences in model recommendations based solely on patient sociodemographic characteristics. For instance, cases labeled as Black or unhoused were more likely to receive recommendations for urgent care, mental health referrals, or invasive interventions—despite identical clinical presentations. These disparities raise concerns that AI tools, when trained on historically biased healthcare data, may perpetuate or even amplify existing imperfections in the data. What went wrong is not rooted in malicious algorithms or ill intent. This phenomenon highlights a core principle regarding AI tools: model outputs inherently reflect the structure and biases of, and gaps in, the data used to train them (Rejeleene et al., 2024).

Another interesting study mentioned that the data sources used to develop clinical AI models were affiliated with high income countries or with specific regions. Over half of the databases used to train models came from either the U.S. or China (Celi et al., 2022). Repeatedly feeding models with data that lack diversity i.e. poorly - represented populations and often curated from restricted clinical settings, can severely limit the generalizability of results and yield biased AI-based decisions (Futoma, et al., 2020).

Other studies show a number of socio-cultural factors that affect patient behaviors and decisions making, contribute towards non-compliance to drugs, and eventually health outcomes (Oates et al., 2020). Of these factors, cultural and religious beliefs are one of the most recognized (Leporini et al., 2014). For example, a study based on review of empirical articles (Brown et al., 2022), highlighted how the cultural and religious beliefs of Jamaicans about pharmacotherapy could be a significant contributor to poor adherence rates in patient living with non-communicable diseases.

These studies raise concern about the adoption and applicability of AI models (trained on data from specific regions) in countries that operate under different medical and healthcare structures.

In healthcare, where data are generated within systems shaped by structural disparities and missing information, the consequences are especially acute. It can shape how AI systems and tools see patients and more critically, how they do not see them. This underscores a critical risk for the increasing proposals to use AI in pharmacovigilance (PV) (Sahni and Carrus, 2023): when key information is missing or unevenly represented, AI-driven tools may fail to detect important safety signals or point out spurious ones. It may also propagate those distortions into downstream assessments of causality and regulatory actions.

## Implications of using AI systems in pharmacovigilance

While AI is transforming key areas of drug development (Li et al., 2025; Zhu and Ouyang, 2022) including target identification, clinical trial optimization, and real-world evidence generation, it also might introduce critical vulnerabilities, particularly through its amplification of pre-existing biases rooted in missing or incomplete data. In PV, where the stakes are high and decisions must reflect nuanced clinical and demographic realities, such biases can compromise both the detection of safety signals and subsequent causality assessments.

Drug safety signal detection depends, particularly in the post-marketing setting, on the ability to identify emerging risks from large volumes of real-world data where diverse populations and long-term outcomes come into focus. However, biased, missing or incomplete patient data can significantly distort this process, where early signal detection relies on recognizing subtle but meaningful patterns across diverse patient groups. Unequal access to care among low-income, rural, or marginalized communities results in fewer documented interactions with the healthcare system, making these groups underrepresented in Electronic Health Records (EHRs) and spontaneous reports. For example, social risk factors such as housing instability, domestic violence, or mental health struggles—are routinely underreported or omitted altogether in clinical documentation (Cantor and Thorpe, 2018). These missing contextual details can critically affect both drug response and safety profiles. Underreporting of risk factors that might play a confounding or effecting modifying role, further narrows the context needed to assess safety concerns.

The effectiveness of AI-driven signal detection depends not only on the volume of data available but also on the completeness and representativeness of that data across populations. When real-world data sources such as EHRs, insurance claims, or spontaneous adverse event reports—are unevenly distributed, AI models are more likely to favor well-documented groups while overlooking or misclassifying risks in underrepresented populations. For example, individuals from communities with limited access to healthcare or low trust in medical institutions may report fewer ADRs due to linguistic, cultural, or socioeconomic barriers. This underreporting can lead to false assumptions of safety in these groups. Similarly, when clinical trials lack demographic diversity, early warning signs of subgroup-specific risks may go undetected. Certain adverse events are known to occur more frequently in specific racial or genetic populations for instance, severe cutaneous reactions associated with HLA-B*1502 are significantly more common in East Asian patients (Chen et al., 2011). If such subgroups are underrepresented in the data used to train AI systems, critical safety concerns may be missed, delaying updates to product labels, prescribing guidelines, and risk mitigation strategies.

Moreover, clinician documentation practices can reflect implicit biases, with varying levels of detail or emphasis depending on the patient’s background (Sabin, 2022). The fragmented nature of health data across different care settings also limits the completeness of patient histories. Regulatory constraints, though essential for privacy, may further impede access to attributes like race or socioeconomic status—precisely the variables needed to detect and mitigate bias. Together, these issues might result in distorted or incomplete safety signals. Compounding this problem, AI models trained on biased data may appear to perform well when evaluated globally but fail in underrepresented subgroups. For example, a model may demonstrate high specificity—correctly identifying true negatives in majority populations—while exhibiting low sensitivity in detecting true positives among minorities. This imbalance might create a false sense of model reliability and masks risk precisely where it is most likely to go undetected (Obermeyer et al., 2019).

When signals are distorted at the detection stage, the downstream impact on causality assessment can be profound. Causality assessment relies not only on the signal itself but on a comprehensive understanding of case-level detail, confounding variables, and background incidence rates as well as many other streams of evidence. The decision-making already involves complex probabilistic reasoning (Hammad et al., 2023; Hammad and Davies, 2025) and using AI system with missing data can obscure key temporal associations, omit co-medications or comorbidities, and reduce the ability to apply structured algorithms or clinical judgment with confidence. This, in turn, can lead to delayed or incorrect conclusions about a product’s benefit risk profile either failing to act when necessary or acting on misleading information, which could divert resources or erode trust.

## Discussion

Successful integration of AI into PV workflows requires more than algorithmic sophistication. It demands a deliberate focus on equity, transparency, and contextual relevance. Missing data must not be treated as a minor technical nuisance. Rather it is a driver of analytic misjudgment and a potential source of harm. Addressing this challenge calls for systematic bias auditing, tailored model calibration, and governance structures that place patient safety at the forefront.

While the challenges posed by missing data and bias in AI-driven signal detection and causality assessment are serious, they are not insurmountable. Acknowledging the problem is the first step; the next is to take meaningful action across multiple fronts. If the promise of AI in PV is to be fully realized, we must invest in coordinated action across data infrastructure, modeling approaches, and workforce development. Improving the quality and completeness of claim, EHRs, and real-world data should be prioritized. This includes training staff in consistent documentation practices and promoting interoperable data-sharing frameworks across healthcare systems and insurers. Although such systemic changes are time-consuming, they are necessary to ensure that all patients are represented in the data used for drug safety evaluation. The question is whether the rapid pace of AI adoption in drug safety can afford to wait. In the shorter term, AI and statistical methods should be adapted to better handle missing data and adjust for known biases.

Regulators are increasingly aware of these challenges and already taking steps in the right direction. FDA and EMA frameworks for real-world evidence^1^ ^,^ ^2^ emphasize the importance of data completeness, transparency, and bias mitigation. FDA’s guidance on AI and machine learning in drug development^3^ calls for rigorous documentation, ongoing monitoring, model validation, and ethical safeguards to ensure AI use supports patient safety. Guardrails such as model explainability, independent audits, and human review—are critical to ensuring that AI complements rather than compromises PV (Wiens et al., 2019).

Lastly, a prepared workforce is critical (Hammad et al., 2023). PV professionals, data scientists, and clinicians must be equipped to recognize the limitations of AI models and interpret outputs in context. Educational initiatives, including organization-sponsored training on AI bias and data equity, should be integral to any implementation strategy. Online platforms like Coursera and LinkedIn Learning offer relevant training programs, and companies should consider sponsoring staff participation as part of responsible adoption planning. AI can be one of the most powerful tools in drug safety, but only if we ensure it sees the full picture. The promise of AI in PV hinges on our ability to teach it to see the whole picture; garbage-in truly is garbage-out. We must commit to building systems in which no patient—and no safety signals are left out of the data that drives tomorrow’s PV.

## Footnotes

1
https://www.fda.gov/science-research/science-and-research-special-topics/real-world-evidence

2 EMA’s Guidance: Journey towards a roadmap for regulatory guidance on real-world evidence (2025) https://www.ema.europa.eu/en/documents/other/journey-towards-roadmap-regulatory-guidance-real-world-evidence_en.pdf

3 FDA’s Center for Drug Evaluation and Research (CDER) (2025). Using Artificial Intelligence and Machine Learning in the Development of Drug and Biological Products https://www.fda.gov/about-fda/center-drug-evaluation-and-research-cder/artificial-intelligence-drug-development
